# Elevated Cerebrospinal Fluid Neopterin Concentration Is Associated with Disease Severity in Acute Puumala Hantavirus Infection

**DOI:** 10.1155/2013/634632

**Published:** 2013-07-31

**Authors:** Timo Hautala, Terhi Partanen, Tarja Sironen, Saara-Mari Rajaniemi, Nina Hautala, Olli Vainio, Olli Vapalahti, Heikki Kauma, Antti Vaheri

**Affiliations:** ^1^Department of Internal Medicine, Oulu University Hospital, P.O. Box 20, 90029 Oulu, Finland; ^2^Department of Virology, Haartman Institute, University of Helsinki, P.O. Box 21, 00014 Helsinki, Finland; ^3^Institute of Diagnostics, Department of Medical Microbiology, University of Oulu, 90014 Oulu, Finland; ^4^Department of Ophthalmology, Oulu University Hospital, P.O. Box 21, 90029 Oulu, Finland; ^5^Clinical Microbiology Laboratory, Oulu University Hospital, P.O. Box 50, 90029 Oulu, Finland; ^6^Department of Veterinary Biosciences, Faculty of Veterinary Medicine, University of Helsinki, P.O. Box 66, 00014 Helsinki, Finland; ^7^Department of Virology and Immunology, HUSLAB, Helsinki University Central Hospital, HUS, 00029 Helsinki, Finland

## Abstract

Nephropathia epidemica (NE) caused by Puumala hantavirus (PUUV) is the most common hemorrhagic fever with renal syndrome (HFRS) in Europe. The infection activates immunological mechanisms that contribute to the pathogenesis and characteristics of the illness. In this study we measured cerebrospinal fluid (CSF) neopterin concentration from 23 acute-phase NE patients. We collected data on kidney function, markers of tissue permeability, haemodynamic properties, blood cell count, length of hospitalisation, inflammatory parameters, and ophthalmological properties. The neopterin levels were elevated (>5.8 nmol/L) in 22 (96%) NE-patients (mean 45.8 nmol/L); these were especially high in patients with intrathecal PUUV-IgM production (mean 58.2 nmol/L, *P* = 0.01) and those with elevated CSF protein concentrations (mean 63.6 nmol/L, *P* < 0.05). We also observed a correlation between the neopterin and high plasma creatinine value (*r* = 0.66, *P* = 0.001), low blood thrombocyte count (*r* = −0.42, *P* < 0.05), and markedly disturbed refractory properties of an eye (*r* = 0.47, *P* < 0.05). Length of hospitalisation correlated with the neopterin (*r* = 0.42, *P* < 0.05; male patients *r* = 0.69, *P* < 0.01). Patients with signs of tissue oedema and increased permeability also had high neopterin concentrations. These results reinforce the view that PUUV-HFRS is a general infection that affects the central nervous system and the blood-brain barrier.

## 1. Introduction

Nephropathia epidemica (NE) is an emerging infectious disease caused by Puumala hantavirus carried and spread by bank voles (*Myodes glareolus*) (PUUV) [1]. This hemorrhagic fever with renal syndrome (HFRS) has an acute onset of fever 2–5 weeks after the exposure. The typical symptoms of the acute illness include impaired renal function, gastrointestinal symptoms, thrombocytopenia, blurred vision, and occasionally hemorrhagic complications. Central nervous system (CNS) symptoms are also common; many patients experience headache, insomnia, vertigo, nausea, nuchal rigidity, and confusion. Pituitary haemorrhage, generalized seizures, meningoencephalitis, and acute disseminated encephalomyelitis have been reported [1]. Despite these potential complications, in general the course of the illness is mild and most patients recover without suffering any long-term complications. 

Endothelial cells and monocytes are thought to be the primary targets of hantaviruses. The infection may lead to activation of monocyte/macrophage lineage and expression of interleukin-6 (IL-6), interleukin-10 (IL-10), and tumour necrosis factor-alpha (TNF-*α*) the serum levels of which have been correlated with the severity of the infection [2–4]. It seems evident that immune-mediated mechanisms are at least partly responsible for the symptoms of the illness; activation of monocytes/macrophages followed by cytokine production, complement activation, and a T-cell response may contribute to the pathogenesis of HFRS [1]. Increased vascular permeability and capillary leakage in acute NE may be of immunological origin. In addition, there is a genetic susceptibility to severe disease in individuals with HLAB8DR3 haplotype [5]. 

In the present study, we investigated a marker of monocyte/macrophage lineage activation in the CNS during acute NE. We analyzed the concentrations of neopterin, a pro-inflammatory factor produced mainly by macrophages, in CSF samples of acute-phase NE patients and then searched for associations between the CSF neopterin levels and other clinical characteristics of the illness.

## 2. Materials and Methods

### 2.1. Patient Population

The patient population of this study was described in detail elsewhere [6]. The CSF sample was collected from 42 of the 58 patients. For this analysis, we included 23 hospitalised NE patients from whom adequate acute-phase CSF samples were available for the determination of neopterin concentration. The study protocol was explained to each patient and they had the right to participate, refuse, or withdraw from the study or any single examination of the study according to the Helsinki Declaration. The ethics Committee of the Oulu University Hospital approved the study and all participants signed an informed consent form. 

### 2.2. Clinical Data

Patients were examined as previously described [6]. The patients' CNS symptoms were evaluated and CSF samples were collected. Data of kidney function (daily urine output and plasma creatinine), tissue permeability and tissue oedema (serum/CSF albumin ratio, weight change during hospitalisation), and haemodynamic parameters (systolic blood pressure, heart rate) were collected. The length of hospitalisation as a marker of disease severity was recorded. In addition, blood haemoglobin (Hb) concentration, thrombocyte count, blood white cell count (BWC), serum albumin concentration, plasma C-reactive protein (CRP) concentration, CSF glucose, protein and albumin concentrations, and CSF white cell count were determined. The patients were also examined by an ophthalmologist as described earlier [7]. In addition, the previously described results of brain magnetic resonance imaging (MRI), electroencephalography (EEG), and human leukocyte antigen (HLA) haplotyping were considered [6].

### 2.3. Virological and Immunological Analysis

The laboratory diagnosis of NE was based on PUUV serology that was initially analysed using a commercial enzyme-linked immunosorbent assay of IgM antibodies (Reagena Puumala IgM EIA Kit, Reagena, Toivala, Finland). In selected cases, the samples were also analysed with an indirect immunofluorescence test for PUUV-IgG which displayed a granular staining pattern in cases of typical acute infection [8]. CSF and serum PUUV IgM and IgG antibody levels were titrated as described earlier [6]. The CSF neopterin concentration was analysed using a commercial enzyme-linked immunosorbent assay (Neopterin ELISA, IBM International GMBH, Hamburg, Germany). Neopterin concentrations were calculated from optical density values of duplicate samples using cubic spline interpolation with a not-a-knot condition (http://www.akiti.ca/CubicSpline.html).

### 2.4. Statistical Methods and Data Analysis

Multivariate analysis of the results was not possible due to the low number of patients. Instead, Pearson bivariate correlation test and Mann-Whitney *U* test were used to analyse the significance of the findings. A *P* value of <0.05 was considered significant. In the case of ophthalmological parameters, the values measured from the right eye of each patient were used. PASW Statistics 18 (SPSS Inc., Chicago, IL, USA) was used for statistical analysis.

## 3. Results

### 3.1. CSF Neopterin

CSF samples from 23 patients with acute NE were available for analysis of the neopterin concentration. Most (*n* = 22, 96%) patients had an elevated CSF neopterin level compared to those seen in healthy individuals (<5.8 nmol/L) [9, 10]. The CSF neopterin mean concentration in male (*n* = 15) and female (*n* = 8) patients was 46.9 nmol/L and 43.9 nmol/L, respectively. The neopterin concentration was 63.6 nmol/L and 29.6 nmol in CSF in samples with elevated (>500 mg/L, *n* = 11) or normal (150–500 mg/L, *n* = 12) protein level, respectively (*P* < 0.05, Mann-Whitney *U* test) (Table 1). Patients with a positive CSF PUUV-IgM finding (*n* = 14) had higher CSF neopterin level (58.2 nmol/L) than those negative for CSF PUUV-IgM (26.5 nmol/L, *n* = 9, *P* = 0.01). The CSF white cell count did not show any association with the neopterin level and there was no difference in the CSF neopterin levels of different age categories. CSF neopterin level correlated with the length of hospitalisation (*r* = 0.42, *P* < 0.05) (Table 2); this finding was most evident among the male patients (*r* = 0.69, *P* < 0.01).

### 3.2. CSF Neopterin and Blood Cell Count

There was a correlation between the blood thrombocyte level measured in the morning of the lumbar puncture and the CSF neopterin concentration (*r* = −0.42, *P* < 0.05) (Table 2). In addition, the blood Hb concentration in the morning of the lumbar puncture was lower in those patients with a high CSF neopterin concentration (*r* = −0.44, *P* < 0.05). There was no association between the blood white cell count and the CSF neopterin level. 

### 3.3. Kidney Function, Tissue Oedema, and Permeability

The highest plasma creatinine level (*P* < 0.01) and the plasma creatinine concentration measured in the morning of the lumbar puncture (*r* = 0.66, *P* = 0.001) correlated with the CSF neopterin concentration (Table 2). Weight change during the hospitalisation correlated with the CSF neopterin concentration (*r* = −0.53, *P* < 0.05) indicating that patients with the most evident tissue oedema had the highest CSF neopterin level. CSF/serum albumin ratio as a marker of tissue permeability was also associated with CSF neopterin concentration (*P* = 0.01).

### 3.4. Ophthalmological Findings and CSF Neopterin

An elevated CSF neopterin concentration was associated with a large difference in the refractory properties of the eye when this was first measured during the acute phase and then month after recovery (*r* = 0.47, *P* < 0.05) (Table 2). None of the other ophthalmological parameters were linked with the CSF neopterin measurement. 

### 3.5. Other Clinical Examinations

The patients were analyzed for brain MRI, EEG, and HLA haplotype and the results are described in detail in our previous paper [6]. The number of patients or number of abnormal findings was too low to allow statistical analysis.

## 4. Discussion

An elevated CSF protein concentration and a raised white cell count are commonly seen in acute NE and many patients display evidence of intrathecal antibody production against PUUV [6, 11, 12]. In addition, PUUV RNA has been detected in the pituitary gland and the CSF [13, 14]. A previous study has also confirmed elevated levels of neopterin, IL-6, and interferon-gamma in the CSF of acute NE patients [12]. Based on these data, it seems evident that the CNS is affected in acute NE. However, it remains partly unclear whether the clinical symptoms and the laboratory findings originate from the direct viral invasion of the brain or from increased tissue permeability due to activation of the immune system. 

Activation of monocyte/macrophage lineage and subsequent cytokine expression leading to T-lymphocyte stimulation may contribute to the pathogenesis of acute NE [15]. The intensity of activation of these cells has been shown to account for the course of the disease in individual patients [2–4]. In our patient population, the CNS disease in acute NE appeared to be dependent on these similar inflammatory pathways. In addition, it can be concluded from our current results that the activity of the same pathways may also play a role in NE ocular involvement. The CSF neopterin level was high in our patients with intrathecal PUUV antibody production and those with high CSF protein concentration. An analogous observation has been documented in the occurrence of permanent neurological complications, and even death, in human herpes virus (HSV) encephalitis [16]. In addition, elevated CSF neopterin concentrations have also been detected in other CNS manifestations of viral infections, for example, acute human immunodeficiency virus (HIV) infection and chronic HIV encephalopathy [10, 17].

Elevated neopterin levels have been detected in acute dengue fever and high neopterin levels were found especially in those dengue patients with increased tissue permeability [18]. A similar observation has been made in HIV infection in which a high neopterin concentration indicated increased blood-brain barrier (BBB) permeability [19]. Neopterin has also been shown to play a role in the increased permeability of the BBB in patients with cerebral small vessel disease [20]. In our patient cohort, we detected an association between the CSF neopterin concentration and the weight change during the hospitalisation. We also observed a correlation between the CSF neopterin and the CSF/serum albumin ratio. These results indicate that the inflammatory process with activated monocytes/macrophages may play a role in the increased tissue permeability also in acute NE. Further conclusions regarding the mechanism of increased tissue permeability based on our results are not warranted; a major limitation of our study is that we were not able to examine additional inflammatory markers from both CSF and blood due to low sample volumes. Future studies should be designed to evaluate broad range of indicators of activated pathways from multiple locations. 

In our study, high neopterin levels correlated with markers of disease severity: high plasma creatinine concentrations and low blood thrombocyte counts were encountered in patients with high neopterin values. Moreover, the elevated CSF neopterin level correlated with the length of hospitalisation especially in male patients. Interestingly, a previous study has found a gender-dependent difference in the cytokine responses to hantavirus infection [21]. It is also known that the male gender is associated with a more severe clinical course of NE and young male patients are at a special risk of developing serious CNS complications during acute NE [22].

In summary, it seems possible that an inflammatory reaction of the CNS in acute NE is at least partly influenced by the monocyte/macrophage activity. Increased tissue permeability due to inflammatory reaction is most likely essential, but it is also possible that direct PUUV invasion of the CNS may play a significant role. Investigation of mechanisms leading to the injury of the blood-brain barrier and the increased tissue permeability, however, may further explain the biology of CNS disease in acute NE.

## Figures and Tables

**Table 1:** Summary of statistical analysis of the CSF neopterin concentration and the CSF PUUV IgM, protein concentration, white cell count, and CSF/serum albumin ratio.

	PUUV IgM		CSF protein (mg/L)		CSF white cells (×10*e*6/L)		CSF/serum albumin	
	−	+	150–500	>500	0–3	>3	<0.1	≥0.1
CSF neopterin (nmol/L)	26.5	58.2	29.6	63.6	42.8	50.5	32.2	68.6
Significance	*P* = 0.01		*P* < 0.05		ns		*P* = 0.01	

**Table 2:** Summary of the statistical correlation between the CSF neopterin concentration and plasma creatinine and blood thrombocyte count measured in the morning of lumbar puncture, duration of hospitalisation, weight change during the hospitalisation, and change in the refractory properties of an eye between the acute-phase and one month later.

	CSF neopterin
Plasma creatinine	*r* = 0.66 *P* = 0.001
Blood thrombocytes	*r* = −0.42 *P* < 0.05
Length of hospitalization	*r* = 0.42 *P* < 0.05
Weight change	*r* = −0.53 *P* < 0.05
Change in refraction of eye	*r* = 0.47 *P* < 0.05

## References

[1] Vaheri A, Henttonen H, Voutilainen L (2013). Hantavirus infections in Europe and their impact on public health. *Reviews in Medical Virology*.

[2] Mäkelä S, Mustonen J, Ala-Houhala I (2004). Urinary excretion of interleukin-6 correlates with proteinuria in acute Puumala hantavirus-induced nephritis. *American Journal of Kidney Diseases*.

[3] Outinen TK, Mäkelä SM, Ala-Houhala IO (2010). The severity of Puumala hantavirus induced nephropathia epidemica can be better evaluated using plasma interleukin-6 than C-reactive protein determinations. *BMC Infectious Diseases*.

[4] Saksida A, Wraber B, Avšič-Županc T (2011). Serum levels of inflammatory and regulatory cytokines in patients with hemorrhagic fever with renal syndrome. *BMC Infectious Diseases*.

[5] Mustonen J, Partanen J, Kanerva M (1996). Genetic susceptibility to severe course of nephropathia epidemica caused by Puumala hantavirus. *Kidney International*.

[6] Hautala T, Mhnen S-M, Sironen T (2010). Central nervous system-related symptoms and findings are common in acute Puumala hantavirus infection. *Annals of Medicine*.

[7] Hautala N, Kauma H, Vapalahti O (2011). Prospective study on ocular findings in acute Puumala hantavirus infection in hospitalised patients. *British Journal of Ophthalmology*.

[8] Vaheri A, Vapalahti O, Plyusnin A (2008). How to diagnose hantavirus infections and detect them in rodents and insectivores. *Reviews in Medical Virology*.

[9] Berdowska A, Zwirska-Korczala K (2001). Neopterin measurement in clinical diagnosis. *Journal of Clinical Pharmacy and Therapeutics*.

[10] Hagberg L, Cinque P, Gisslen M (2010). Cerebrospinal fluid neopterin: an informative biomarker of central nervous system immune activation in HIV-1 infection. *AIDS Research and Therapy*.

[11] Alexeyev OA, Morozov VG (1995). Neurological manifestations of hemorrhagic fever with renal syndrome caused by Puumala virus: review of 811 cases. *Clinical Infectious Diseases*.

[12] Ahlm C, Linden C, Linderholm M (1998). Central nervous system and ophthalmic involvement in nephropathia epidemica (European type of haemorrhagic fever with renal syndrome). *Journal of Infection*.

[13] Hautala T, Sironen T, Vapalahti O (2002). Hypophyseal hemorrhage and panhypopituitarism during Puumala virus infection: magnetic resonance imaging and detection of viral antigen in the hypophysis. *Clinical Infectious Diseases*.

[14] Mähönen S-M, Sironen T, Vapalahti O (2007). Puumala virus RNA in cerebrospinal fluid in a patient with uncomplicated nephropathia epidemica. *Journal of Clinical Virology*.

[15] Sadeghi M, Eckerle I, Daniel V, Burkhardt U, Opelz G, Schnitzler P (2011). Cytokine expression during early and late phase of acute Puumala hantavirus infection. *BMC Immunology*.

[16] Bociaga-Jasik M, Ciesla A, Kalinowska-Nowak A, Skwara P, Garlicki A, Mach T (2011). Role of IL-6 and neopterin in the pathogenesis of herpetic encephalitis. *Pharmacological Reports*.

[17] Valcour V, Chalermchai T, Sailasuta N (2012). Central nervous system viral invasion and inflammation during acute HIV infection. *Journal of Infectious Diseases*.

[18] van de Weg CAM, Koraka P, van Gorp ECM (2012). Lipopolysaccharide levels are elevated in dengue virus infected patients and correlate with disease severity. *Journal of Clinical Virology*.

[19] Andersson LM, Hagberg L, Fuchs D, Svennerholm B, Gisslén M (2001). Increased blood-brain barrier permeability in neuro-asymptomatic HIV-1-infected individuals—correlation with cerebrospinal fluid HIV-1 RNA and neopterin levels. *Journal of NeuroVirology*.

[20] Rouhl RP, Damoiseaux JG, Lodder J (2012). Vascular inflammation in cerebral small vessel disease. *Neurobiology and Aging*.

[21] Klingström J, Lindgren T, Ahlm C (2008). Sex-dependent differences in plasma cytokine responses to hantavirus infection. *Clinical and Vaccine Immunology*.

[22] Hautala T, Hautala N, Mähönen S-M (2011). Young male patients are at elevated risk of developing serious central nervous system complications during acute Puumala hantavirus infection. *BMC Infectious Diseases*.

